# Washing

**Published:** 1882-08

**Authors:** 


WASHING.

Any man will feel more like a man, in the consciousness of having on a clean shirt; and as the hired help who go through the motions of washing our clothing without accomplishing the object, one-half are particularly disposed to shirk labor in these days of hot suns and suffocating atmospheres, it might be well to adopt some plan by which the same amount of labor expended in half washing our clothing could be made to do the thing thoroughly. Such a plan would be of incalculable value to that army of hard working wives whose husbands are tillers of the soil.

That excellent paper, the OHIO CULTIVATOR, comes to the aid of farmers' wives right gallantly and assures them that the washing may almost do itself while the poor tired woman is lying on the lounge looking at it, thus: Take one pound of sal-soda (saleratus or bi-carbonate of soda, all of which are different names for the same article) and a half pound of unslacked lime, put them in a gallon of water and boil twenty minutes, let it stand till cool, then drain off, put in a small jug or jar; soak your dirty clothes over night, or until they are wet through, then wring them out and rub on plenty of soap, and in one boiler of clothes well covered with water, add one teacupful of washing fluid; boil half an hour briskly, then wash them thoroughly through one suds, rinse, and your old clothes will look better than the old way of washing twice before boiling.

